# Cell Structure Regulation of Polypropylene/Ethylene-Propylene Rubber Bead Foams and Enhanced Mechanical Properties of Their Molded Products

**DOI:** 10.3390/polym18121540

**Published:** 2026-06-21

**Authors:** Zi’ang Hu, Xiulu Gao, Yichong Chen, Jiacheng Wang, Ling Zhao, Dongdong Hu

**Affiliations:** State Key Laboratory of Chemical Engineering and Low-Carbon Technology, Shanghai Key Laboratory of Multiphase Materials Chemical Engineering, School of Chemical Engineering, East China University of Science and Technology, Shanghai 200237, China; y30230089@mail.ecust.edu.cn (Z.H.); gaoxl@ecust.edu.cn (X.G.); chenyc@ecust.edu.cn (Y.C.); y82240088@mail.ecust.edu.cn (J.W.); zhaoling@ecust.edu.cn (L.Z.)

**Keywords:** polypropylene, ethylene-propylene rubber, bead foaming, mechanical properties

## Abstract

To improve the foamability and steam-chest molding performance of polypropylene (PP) bead foams, ethylene-propylene rubber (EPR) was introduced into PP via melt blending. The role of EPR in the complete bead-foaming-to-molding process was systematically investigated by correlating phase morphology, crystallization behavior, melt viscoelasticity, CO_2_ dissolution and diffusion, cellular structure, inter-bead welding, and the mechanical properties of molded foam products. The incorporation of EPR refined the PP crystalline morphology, reduced the apparent crystallinity, and markedly enhanced the melt viscoelasticity, thereby broadening the foaming temperature window. The dispersed EPR phase functioned simultaneously as a CO_2_ reservoir and a high-diffusivity pathway of CO_2_, which promoted cell growth while suppressing excessive nucleation. The enhanced melt viscoelasticity and improved CO_2_ affinity promoted bead expansion and optimized the cellular structure. At 150 °C, the expansion ratio increased from 18.7 for neat PP to 21.1 with 10 wt% EPR. EPR also regulated the cellular structure. At 150 °C, the cell diameter increased from 83 to 176 μm as the EPR content increased from 0 to 20 wt%. EPR markedly changed the double-melting behavior of PP bead foams. The low-temperature melting enthalpy increased from 28.5 J/g for neat PP to 37.8 J/g with 10 wt% EPR, which served as an effective interfacial binder, significantly promoting inter-bead welding. Consequently, the optimized PP/EPR foam containing 10 wt% EPR exhibited a tensile strength of 1.13 MPa and an elongation at break of 22.1%. More importantly, excellent molding quality was achieved at a reduced steam pressure of 2.2 bar, demonstrating the great potential of PP/EPR bead foams for the energy-efficient manufacturing of high-performance lightweight products.

## 1. Introduction

Polypropylene (PP) has been widely utilized in automotive manufacturing, packaging logistics, and building insulation owing to its low density, excellent chemical resistance, high heat deflection temperature, and good recyclability [1,2]. In recent years, driven by the increasing demand for metal replacement and lightweight components, high-performance PP foams, particularly expanded polypropylene (EPP) beads, have received growing research interest [3]. However, conventional PP possesses a highly linear molecular architecture, which gives rise to insufficient melt strength. As a result, it is difficult for PP melts to withstand the biaxial extensional stresses generated during cell growth, frequently resulting in cell coalescence, collapse, and consequent deterioration of the cellular structure. This limitation restricts the fabrication of low-density PP foams with high expansion ratios [4]. Furthermore, the high crystallinity and rapid crystallization rate of PP result in a narrow foaming temperature window. Together with its tendency toward brittle fracture at low temperatures, these drawbacks further hamper the use of PP foams in high-end applications [5].

Among commercially used polymer foams, polyurethane (PU) foams are widely applied in cushioning, thermal insulation, furniture, and automotive interiors because of their highly tunable cellular structures and broad property range [6,7]. However, many conventional PU foams are chemically crosslinked thermoset materials, which limits their melt reprocessing and mechanical recycling [8]. In contrast, PP-based bead foams are thermoplastic polyolefin foams and are therefore attractive for recyclable lightweight molded products [9]. In this context, the potential users of PP/EPR bead foams are manufacturers of protective packaging, reusable logistics components, automotive cushioning and energy-absorbing parts, and lightweight molded structures, where low density, chemical resistance, heat resistance, recyclability, and sufficient inter-bead welding strength are required. Therefore, PP/EPR bead foams should be regarded as complementary to PU foams rather than as direct substitutes for all PU foam applications.

To address these limitations, various modification strategies have been developed, including long-chain branching, cross-linking, nanoparticle incorporation, and polymer blending [10,11,12]. Although long-chain branching and cross-linking can effectively enhance the melt strength of PP, they are often associated with complex processing behavior and reduced recyclability. By contrast, melt blending has been widely adopted because of its simple processing procedure, cost-effectiveness, and suitability for industrial scale-up [13,14]. Among different blending components, elastomers such as ethylene-propylene-diene monomer (EPDM) and polyolefin elastomer (POE) have been extensively used to improve the toughness and low-temperature impact resistance of PP. Moreover, these elastomeric phases can regulate the rheological behavior and crystallization kinetics of the PP matrix by forming multiphase structures [2,11].

The introduction of a rubber phase has a complex influence on the foaming behavior of PP. Well-dispersed rubber particles can serve as heterogeneous cell-nucleation sites during foaming, thereby reducing the energy barrier for bubble nucleation and regulating cell density [15,16,17]. However, owing to the larger free volume of the rubber phase and its stronger affinity for CO_2_, EPR can alter CO_2_ solubility and diffusion within the polymer matrix, consequently affecting cell growth and stabilization [16]. For EPP bead foaming, which generally involves pre-foaming followed by steam-chest molding, the crystallization and melting behavior of the material, particularly the formation of a double-melting peak, directly determines the inter-bead welding quality and the mechanical performance of the final molded products [18,19,20]. Nevertheless, most existing studies have focused mainly on the static characterization of elastomer-toughened PP systems. A systematic understanding is still lacking regarding how ethylene-propylene rubber (EPR) dynamically regulates the coupled processes of CO_2_ diffusion, melt crystallization, and cell growth, as well as its specific contribution to inter-bead welding during steam-chest molding. To address this knowledge gap, this study systematically investigates the evolution of phase morphology, CO_2_ dissolution and diffusion behavior, and double-melting characteristics of PP/EPR blends, thereby establishing their quantitative relationships with the cellular structure and mechanical properties of molded bead foam products.

In this study, PP/EPR composites with varying EPR contents were prepared by melt blending to clarify the role of EPR in the complete bead-foaming-to-molding process. Unlike previous studies that mainly focused on elastomer toughening or the one-step foaming behavior of PP blends, this work systematically correlates EPR-induced phase morphology evolution with CO_2_ desorption kinetics, melt viscoelasticity, bead foaming behavior, double-melting characteristics, inter-bead welding, and the mechanical properties of molded foam products. The novelty of this work is that EPR is not treated only as a rubber toughening phase, but as a multifunctional phase that simultaneously regulates CO_2_ transport, cell growth, melt stabilization, and bead-interface bonding. These findings provide a mechanistic basis for optimizing PP/EPR bead foams with broadened processing windows and improved molded-product performance.

## 2. Materials and Methods

### 2.1. Materials

Polypropylene (PP, 6622PT) was supplied by SABIC, Riyadh, Saudi Arabia, with a density of 0.905 g/cm^3^ and a melt index of 8.0 g/10 min at 230 °C/2.16 kg. Ethylene-propylene rubber (EPR, Vistalon 722) was obtained from ExxonMobil, TX, USA, with a density of 0.865 g/cm^3^ and a melt index of 1.0 g/10 min at 190 °C/2.16 kg. The antioxidant Irganox 1010 was purchased from BASF, Ludwigshafen, Germany. CO_2_ and N_2_, both with a purity of 99.995%, were provided by Shanghai Air Liquide, Shanghai, China. All materials were used as received.

### 2.2. Preparation of PP/EPR Blends

PP/EPR blends were prepared using a co-rotating twin-screw extruder (KTE-25D, Nanjing Keerke Extrusion Equipment Co., Ltd., Nanjing, China) at barrel temperatures of 150, 200, 200, 200, 200, and 180 °C and a screw speed of 200 rpm. The extruded strands were then pelletized into micropellets with an average weight of approximately 1.2 mg per pellet. The detailed blend formulations and sample codes are summarized in Table 1.

For bead foaming, 300 g of PP/EPR micropellets, 25 L of deionized water, 30 g of kaolin, and 12 g of sodium benzenesulfonate were charged into a 29 L high-pressure autoclave. Kaolin and sodium benzenesulfonate were used as dispersing agents to prevent adhesion and agglomeration of the micropellets during foaming. The suspension was stirred at 200 r/min, and CO_2_ was introduced into the autoclave. The system was then heated to the selected foaming temperature of 148, 150, or 152 °C and maintained at 2.8 MPa for 30 min to allow sufficient CO_2_ saturation. After saturation, the discharge valve was rapidly opened, and the CO_2_-saturated micropellets were discharged under the pressure difference between the autoclave and the atmosphere, resulting in bead expansion. The obtained EPP beads were collected, dehydrated, and dried at 70 °C for 2 h.

**Figure 1:**
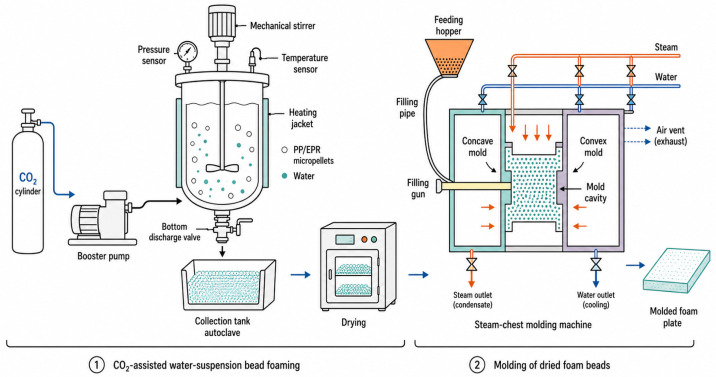
Schematic illustration of the CO_2_-assisted water-suspension bead foaming and steam-chest molding process.

The dried EPP beads were molded into specimen plates with dimensions of 200 mm × 200 mm × 20 mm using a steam-chest molding machine (Mini-888, Jiuh Shin Machinery (Suzhou) Co., Ltd., Suzhou, China). Unless otherwise specified, the molding steam pressure was 2.8 bar. The steam heating and cooling times were 1 min and 2 min, respectively.The CO_2_-assisted water-suspension bead foaming and steam-chest molding process is shown in Figure 1.

### 2.3. Differential Scanning Calorimetry (DSC) Analysis

The melting and crystallization behavior of PP/EPR blends was characterized using a differential scanning calorimeter (DSC, NETZSCH DSC 204HP, Germany) under a nitrogen (N_2_) atmosphere. The samples were first heated from room temperature to 200 °C at a rate of 10 °C/min and held for 5 min to erase their thermal history. They were then cooled to 40 °C at 10 °C/min, followed by a second heating scan from 40 to 200 °C at the same heating rate. The second heating and cooling curves were used to analyze the melting and crystallization behavior of the samples.

### 2.4. Rheological Properties

Small-amplitude oscillatory shear (SAOS) rheological measurements were performed using a HAAKE rheometer (MARS III, USA). All measurements were conducted at 180 °C to ensure complete melting of the PP matrix, and a nitrogen atmosphere was maintained throughout the tests to prevent oxidative degradation. The frequency sweep tests were carried out under a constant shear stress of 25 Pa with a strain amplitude of 1%, over an angular frequency range of 0.1~100 rad/s.

### 2.5. Morphological Characterization

The samples were cryo-fractured in liquid nitrogen to obtain brittle fracture surfaces. The fracture surfaces were then sputter-coated with platinum to enhance electrical conductivity and observed using a scanning electron microscope (SEM, JEOL JSM-6360LV, Japan). The resulting SEM micrographs were analyzed using Image-Pro Plus 6.0 software (Media Cybernetics) to determine the average cell diameter (*D*) and cell density (*N*_0_). For each foaming condition, five foam beads were randomly selected for SEM observation. The mean cell diameter of each bead was first calculated from the cells visible in the corresponding cross-sectional SEM image. The cell diameter reported for each sample represents the average of the bead-level mean cell diameters, and the error bars represent the standard deviation among different foam beads. This statistical method was used to evaluate the bead-to-bead variation in cellular structure.

The average cell diameter (*D*) was calculated using the following equation:

(1) $$D=\frac{\sum D_{i}n_{i}}{\sum n_{i}}$$

where *n_i_* refers to the number of cells with a diameter of *D_i_* in the SEM image.

(2) $$N_{0}={\left(\frac{n}{A}\right)}^{\frac{3}{2}}\times R_{V}$$

where *n* is the total number of cells in the SEM image, *A* is the area of the micrograph, and *R_V_* is the volume expansion ratio of the foam.

### 2.6. Mechanical Properties Test

The mechanical properties of the samples were evaluated using an electronic universal testing machine (CMT4202/ZWICK/Z020, Shanghai Jiehu Instruments and Meters Co., Ltd., Shanghai, China). Unfoamed dumbbell-shaped specimens with dimensions of 50 mm × 4 mm × 2 mm and foamed dumbbell-shaped specimens with dimensions of 120 mm × 10 mm × 10 mm were used for tensile testing. The foamed tensile specimens were prepared using a hot-wire cutter. Tensile tests were conducted at a constant crosshead speed of 500 mm/min. Compression tests were performed on cubic foam specimens with dimensions of 20 mm × 20 mm × 20 mm at a crosshead speed of 5 mm/min. The tensile modulus was calculated from the slope of the initial linear region of the stress–strain curve using the same strain interval for all samples. All mechanical results are reported as mean ± standard deviation, and at least 5 specimens were tested for each condition.

### 2.7. Measurement of CO_2_ Dissolution and Diffusion Behavior

To quantitatively evaluate the CO_2_ diffusion behavior in PP and PP/EPR blends, gas desorption kinetics measurements were performed. The PP and PP/EPR samples with different EPR contents were prepared into circular disk specimens and saturated with CO_2_ at 5 MPa and room temperature for 12 h to ensure complete dissolution equilibrium. The pressure in the autoclave was then slowly released to atmospheric pressure within 2 min to minimize cell nucleation. After depressurization, the specimens were rapidly removed, and their mass was continuously recorded for 30 min at room temperature and atmospheric pressure using a high-precision analytical balance to monitor the mass variation with time.

The desorption diffusion coefficients, *D_d_*, of samples under these conditions were calculated using the following equation:

(3) $$\frac{M_{t}}{M_{0}}=1-\frac{4}{d}{\left(\frac{D_{d}t}{\pi }\right)}^{\frac{1}{2}}$$

where *M_t_* is the gas uptake at time t, *M*_0_ is the equilibrium gas uptake, *d* is the specimen thickness, and *D_d_* is the desorption diffusion coefficient.

Use of generative AI tools: Generative AI was used to assist with English language polishing, grammar checking, response-letter drafting, improvement of textual clarity, and preparation of an editable schematic illustration.

## 3. Results and Discussion

### 3.1. Morphology of PP/EPR Blends

**Figure 2:**
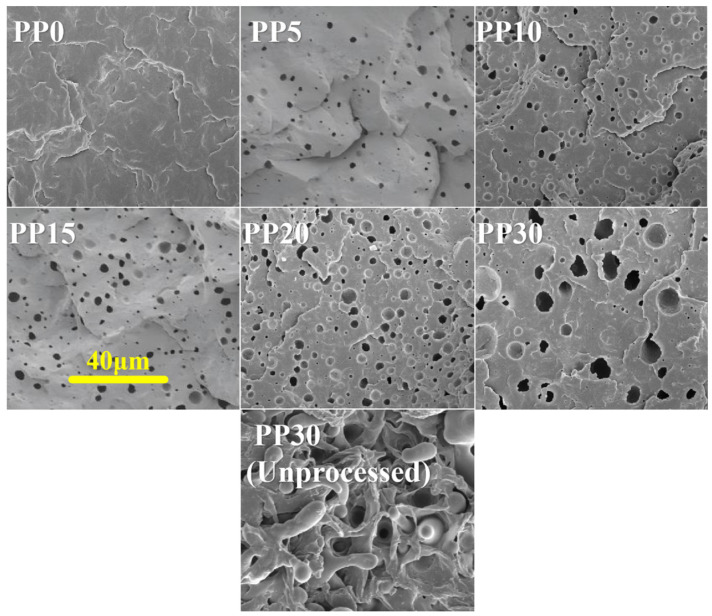
SEM micrographs of the xylene-etched fracture surfaces of PP/EPR blends with different EPR contents.

Figure 2 shows SEM micrographs of the xylene-etched fracture surfaces of the PP/EPR blends. The cavities left by the extracted EPR phase clearly reveal the phase morphology of the blends. At low EPR contents, the EPR domains are relatively uniformly dispersed within the PP matrix, despite a relatively broad domain size distribution.

As the EPR content increases, the average size of the dispersed EPR domains gradually increases, while the interparticle distance decreases. At EPR contents of 5–20 wt%, the blends mainly maintain a sea–island morphology, which is beneficial for subsequent bead foaming because the dispersed EPR domains can serve as CO_2_-rich regions and provide additional interfaces for cell nucleation and growth. Among these compositions, 10–15 wt% EPR provides a more balanced phase morphology, with sufficient dispersed rubber domains while preserving the continuous PP matrix. Therefore, this range was considered a suitable composition window for the following foam preparation.

When the EPR content reaches 30 wt%, the blend begins to transform from a sea–island morphology into a co-continuous structure. The cavities become irregular and serrated, and the EPR domains evolve from spherical particles into elongated and aggregated structures. This morphological transition may cause excessive CO_2_ diffusion, unstable cell growth, and deterioration of mechanical properties, making the 30 wt% EPR blend less suitable for bead foaming.

### 3.2. Non-Isothermal Crystallization and Melting Behavior

**Figure 3:**
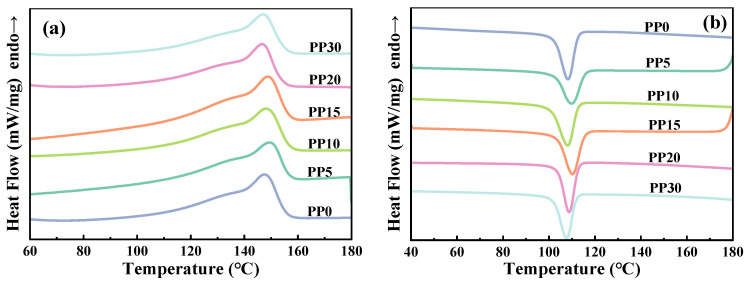
DSC curves of PP and PP/EPR blends: (**a**) melting curves and (**b**) crystallization curves.

To investigate the effect of EPR content on the thermal properties of the PP/EPR blends, DSC analysis was performed on neat PP and the blend samples. As shown in Figure 3a, all samples exhibit a single melting peak during the heating scan, indicating that EPR incorporation does not alter the α-crystalline structure of the PP matrix. The melting peak temperature (*T_m_*) shows only slight fluctuations with increasing EPR content, suggesting that the melting behavior of PP is not significantly affected. In contrast, EPR incorporation markedly influences the crystallization behavior of PP. As summarized in Table 2, the crystallization temperature (*T_c_*) and melting temperature (*T_m_*) show only slight fluctuations with increasing EPR content, indicating that EPR incorporation does not substantially change the crystallization and melting temperatures of the PP phase. The apparent crystallinity (*X_c,app_*), calculated based on the total mass of the PP/EPR blends, decreases slightly from 24.5% for neat PP to 21.0% for the blend containing 30 wt% EPR. This decrease is mainly attributed to the dilution effect of the amorphous EPR phase in the blends.

To further clarify the crystallization behavior of the PP phase, the PP-normalized crystallinity (*X_c,PP_*) was also calculated by considering the PP mass fraction in each blend. The *X_c,PP_* values do not decrease with EPR incorporation, suggesting that the intrinsic crystallization capability of the PP phase is not suppressed. This result may be related to the local nucleation effect of ethylene-rich EPR segments or PP/EPR interfaces. Therefore, EPR incorporation only slightly changes the overall apparent crystallinity of the blends, while the crystallization behavior of the PP phase remains largely preserved.

**Figure 4:**
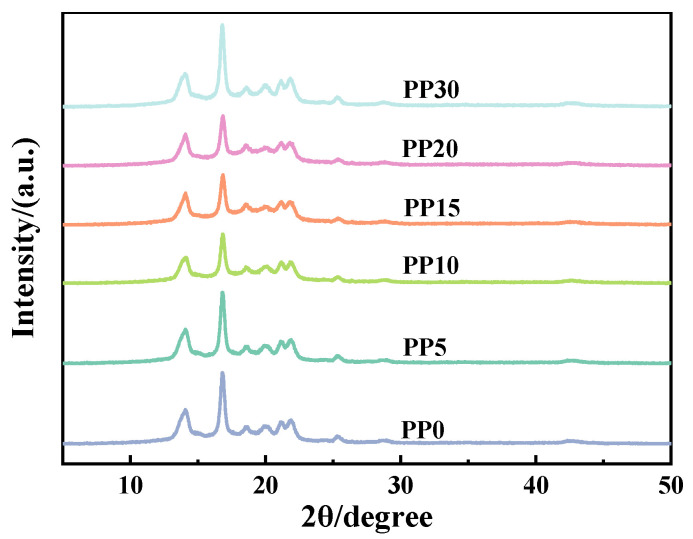
XRD patterns of PP/EPR blends.

Figure 4 shows the X-ray diffraction (XRD) patterns of neat PP and the PP/EPR blends. Neat PP exhibits typical diffraction peaks of the α-crystalline form at 2*θ* ≈ 14.1°, 16.9°, 18.6°, and 21.2°, corresponding to the (110), (040), (130), and (111)/(131) crystal planes, respectively [21,22]. With increasing EPR content, the diffraction peaks exhibit a gradual decrease in intensity and a noticeable broadening, accompanied by a reduction in crystallite size across multiple crystal planes, as summarized in Table 3. These results indicate that EPR incorporation restricts the ordered packing of PP molecular chains, reduces the overall crystallinity, refines the PP crystals, and promotes the formation of imperfect crystals. When the EPR content reaches 30 wt%, the formation of a co-continuous phase structure and the irregular interfaces generated by EPR aggregation may provide supplementary nucleation sites, thereby promoting the growth of certain crystal planes. Furthermore, no distinct characteristic peak of the β-crystalline form (2*θ* ≈ 16°) is observed in the PP/EPR blend systems, demonstrating that EPR addition does not induce a crystal modification transition in PP and that the α-crystalline form remains dominant.

EPR incorporation significantly alters the crystallization behavior of PP. Although it reduces the overall apparent crystallinity of the blends, refines the PP spherulitic structure, and interferes with the formation of perfect crystals, the intrinsic crystallization capability of the PP phase per unit mass is not compromised. Owing to the relatively high ethylene content (72%) of the EPR used in this study, some ethylene segments may act as heterogeneous nucleation sites and facilitate crystallization of the PP phase. During foaming, particularly at temperatures above the melting point of PP, these crystals are largely melted. Therefore, cell growth is not governed solely by the initial crystallinity, but is more directly controlled by the melt rheological properties, which are jointly determined by the molten crystalline structures, EPR molecular chains, and interfacial interactions. The influence of crystallization behavior is mainly reflected in the melting temperature range and the initial melt structure of the blends, thereby affecting the foaming processing window.

### 3.3. Shear Rheological Properties of PP/EPR Blends

**Figure 5:**
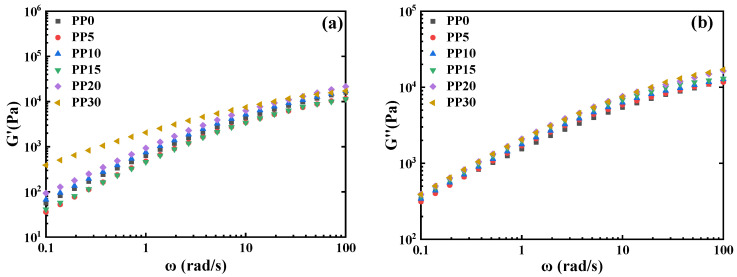
Shear rheological properties of PP/EPR blends at 180 °C: (**a**) storage modulus G′, and (**b**) loss modulus G″.

The viscoelasticity of polymer melts plays a crucial role in cell growth and stabilization during foaming [12]. In this study, small-amplitude oscillatory shear (SAOS) measurements were performed to investigate the rheological properties of PP and PP/EPR blends at 180 °C. Figure 5 shows the storage modulus (G′) and loss modulus (G″) as functions of angular frequency.

After EPR incorporation, the storage modulus shows little change in the high-frequency region but increases markedly in the low-frequency region. The low-frequency rheological response is closely related to the long-time relaxation behavior and phase connectivity of the blends [23]. As shown in Figure 2, EPR is mainly dispersed as rubber domains in the continuous PP matrix at EPR contents of 5–20 wt%. These dispersed EPR domains introduce additional PP/EPR interfaces and restrict the relaxation of neighboring PP chains. With increasing EPR content, the interparticle distance between EPR domains decreases, and the interaction between adjacent rubber domains becomes stronger. As a result, a more pronounced physical entanglement and interfacial interaction network is formed, leading to the increased low-frequency G′ of the PP/EPR blends [23].

Similar to the storage modulus, the loss modulus of the PP/EPR blends also increases after EPR incorporation. The enhanced G″ indicates increased energy dissipation during oscillatory deformation, which can be attributed to the elastomeric nature of EPR, stronger PP/EPR interfacial interactions, and restricted chain relaxation around the dispersed rubber domains [24]. When the EPR content is further increased, the phase morphology begins to evolve toward a co-continuous structure, as observed for the blend containing 30 wt% EPR. This morphology provides a more continuous deformable EPR-rich network, further enhancing the long-time relaxation response of the blend.

Overall, the changes in G′ and G″ are closely associated with the SEM-observed morphology evolution of the PP/EPR blends. The sea–island morphology at moderate EPR contents enhances interfacial friction and physical entanglement while retaining the continuity of the PP matrix, whereas excessive EPR leads to stronger phase connectivity and a tendency toward co-continuity. The enhanced melt viscoelasticity is beneficial for resisting cell wall thinning and suppressing cell collapse during foaming, thereby contributing to the formation of more stable PP/EPR bead foam structures. However, an excessively high EPR content may also promote rapid CO_2_ transport and unstable cell growth, as discussed in the following sections.

### 3.4. Mechanical Properties

**Figure 6:**
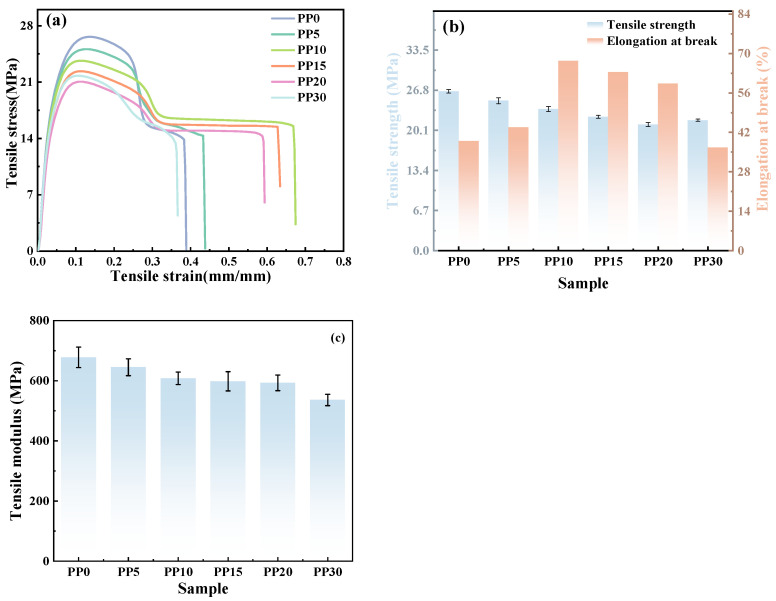
(**a**) Tensile stress–strain curves, (**b**) tensile strength and elongation at break of PP/EPR blends and (**c**) tensile modulus.

The effect of EPR incorporation on the tensile properties of the PP/EPR blends is primarily associated with the evolution of phase morphology and the corresponding variation in deformation mechanisms [24,25,26]. As shown in Figure 6, the tensile modulus gradually decreases with increasing EPR content, from 678 MPa for neat PP to 645, 608, 598, 593, and 536 MPa for the blends containing 5, 10, 15, 20, and 30 wt% EPR, respectively. Although the initial linear portions of the stress–strain curves appear similar, the calculated modulus values confirm a gradual decrease in stiffness after EPR incorporation. This decrease is expected because the soft EPR phase has a lower stiffness than PP and partially reduces the rigidity of the blend.

However, the decrease in tensile modulus is relatively limited at EPR contents of 5–20 wt%. This behavior can be explained by the phase morphology shown in Figure 2. In this composition range, the blends maintain a typical sea–island morphology, in which EPR is dispersed as rubber domains within the continuous PP matrix. Therefore, the initial elastic deformation is still mainly governed by the continuous PP phase and its crystalline framework. The dispersed EPR domains increase the compliance of the blends, but they do not completely interrupt the load-bearing continuity of the PP matrix. As a result, the tensile modulus decreases only moderately in the low-to-medium EPR content range.

At relatively low EPR contents (≤20 wt%), the soft EPR domains act as stress concentrators during tensile deformation, effectively inducing crazing, shear yielding, and plastic deformation in the surrounding PP matrix. A substantial amount of energy is dissipated through these micro-deformation processes, thereby significantly enhancing the macroscopic elongation at break [25]. Nevertheless, because the intrinsic strength of EPR is lower than that of PP, EPR incorporation also dilutes the load-bearing PP phase. Consequently, the tensile strength shows an overall decreasing tendency with increasing EPR content, although the elongation at break is improved in the sea–island morphology region.

When the EPR content increases to 30 wt%, the blend undergoes a distinct morphological transition from a sea–island structure to a co-continuous phase structure. In this morphology, the PP and EPR phases interpenetrate and form continuous networks. As a result, the lower-strength EPR phase also participates in load bearing, leading to a pronounced decrease in the overall tensile strength. More importantly, the co-continuous structure makes the material more prone to crack propagation and localized phase rupture during tensile deformation. Consequently, plastic deformation is restricted, and the energy dissipation mechanism becomes less effective than that in the sea–island morphology. Therefore, the elongation at break sharply decreases to 36.6%, even lower than that of neat PP [26].

### 3.5. Dissolution and Diffusion Behavior of CO_2_

**Figure 7:**
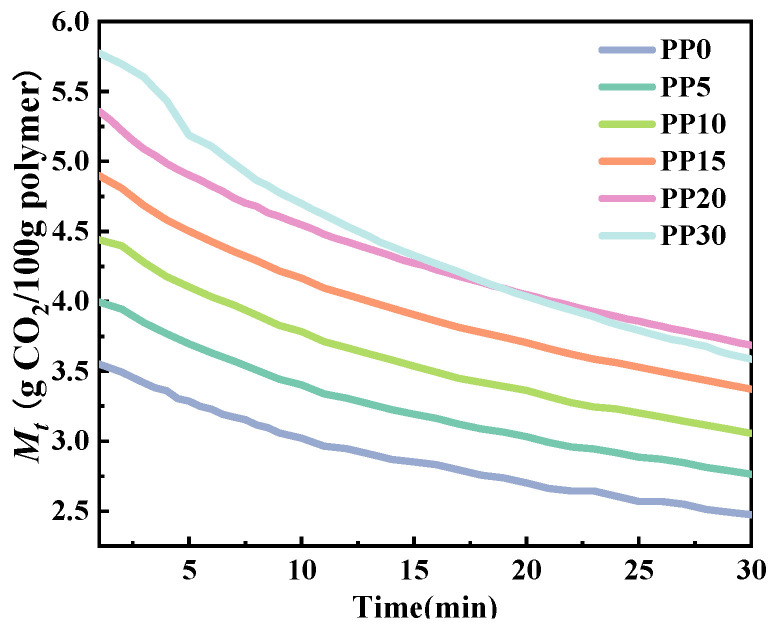
Dissolution and diffusion behavior of CO_2_ in PP and PP/EPR blends.

The dissolution and diffusion behavior of CO_2_ within the polymer matrix is a critical factor governing cell nucleation and growth kinetics during foaming. Figure 7 shows the CO_2_ desorption curves of neat PP and PP/EPR blends with different EPR contents after saturation at 25 °C and 5 MPa. Based on linear fitting of the desorption data, the CO_2_ solubility (M_0_) and desorption diffusion coefficient were calculated, as summarized in Table 4.

The results show that CO_2_ solubility in the blends increases significantly with increasing EPR content. This is mainly attributed to the high free volume of EPR as an amorphous rubber phase, which enhances its affinity for CO_2_ and allows it to serve as a CO_2_ reservoir for subsequent cell growth [27]. Meanwhile, neat PP exhibits the slowest decrease in the desorption curve, indicating the slowest release of dissolved CO_2_ and the lowest gas diffusion coefficient. After EPR incorporation, the CO_2_ desorption rate increases markedly. In particular, within the first 5 min, the amount of desorbed CO_2_ from the PP/EPR blends is much higher than that from neat PP, demonstrating that EPR significantly increases the apparent gas diffusion coefficient of the blends. This behavior can be attributed to the high segmental mobility of EPR, which provides rapid migration pathways for CO_2_ molecules [27].

Notably, when the EPR content reaches 30 wt%, the gas desorption rate increases most significantly. Combined with the phase morphology analysis, this can be ascribed to the formation of a co-continuous structure at this composition. The aggregated and interpenetrating EPR phase forms a continuous diffusion network connected to the external surface, allowing CO_2_ molecules dissolved in the matrix to escape rapidly along this pathway. Such an excessively high gas diffusion rate is unfavorable for stable bead foaming, because the blowing agent tends to dissipate outward through the EPR-rich network rather than diffuse into cell nuclei to support their growth. Consequently, the cellular structure deteriorates.

From the viewpoint of CO_2_ dissolution and diffusion behavior, a moderate EPR content is therefore more suitable for foam preparation. The blends containing 10–20 wt% EPR exhibit enhanced CO_2_ solubility and moderately increased diffusion coefficients, while avoiding the excessive gas loss observed at 30 wt% EPR. Therefore, 10–20 wt% EPR can be regarded as a suitable composition range based on the gas-transport analysis, whereas the final optimized composition should be determined by further considering bead expansion, cellular structure, inter-bead welding, and mechanical properties.

### 3.6. Foaming Behavior of PP/EPR Blends

**Figure 8:**
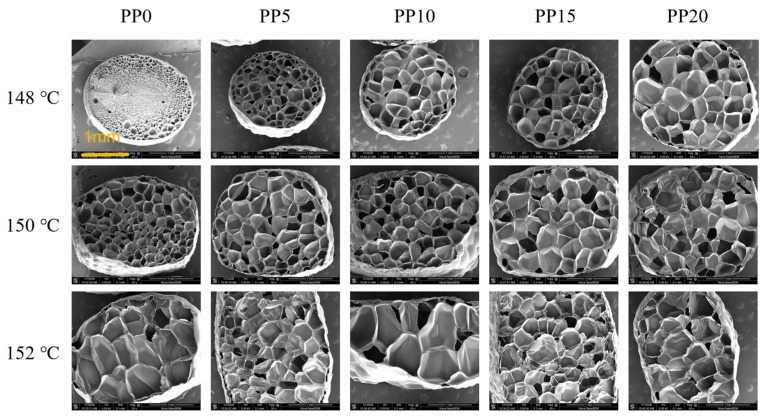
SEM images of PP/EPR bead foams obtained at different foaming temperatures.

To evaluate the effect of EPR content on the bead foaming performance of PP/EPR composites, the evolution of the expansion ratio and cellular structure was investigated at different foaming temperatures. The representative cellular morphologies and the corresponding foaming parameters of PP/EPR bead foams prepared at different foaming temperatures are shown in Figure 8 and Table 5, respectively. At the relatively low foaming temperature of 148 °C, EPR incorporation significantly enhances the expansion ratio, with the foam containing 15 wt% EPR achieving the highest value of 15.3. As the foaming temperature increases to 150 and 152 °C, the expansion ratios of all samples increase substantially. Although the advantage of EPR-modified samples becomes less pronounced at higher temperatures, they still exhibit good foamability over a broader temperature range, indicating that EPR effectively broadens the foaming temperature window.

**Figure 9:**
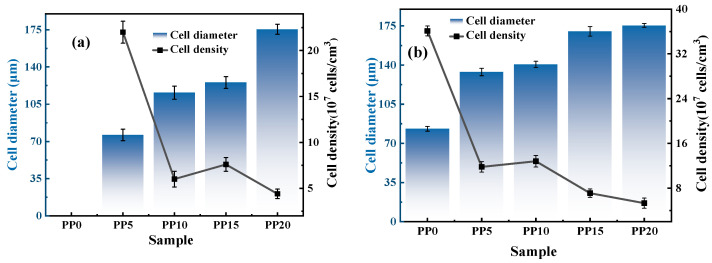
Foaming parameters of PP/EPR bead foams: (**a**) cell diameter and cell density at 148 °C and (**b**) cell diameter and cell density at 150 °C. The error bars of cell diameter represent the standard deviation of bead-level mean cell diameters obtained from different foam beads.

EPR addition also has a pronounced effect on the cellular structure of the bead foams. As shown in Figure 9a,b, at 150 °C, the average cell diameter increases continuously from 83 to 176 μm as the EPR content increases from 0 to 20 wt%, while the cell density decreases from 3.61 × 10^8^ to 5.30 × 10^7^ cells/cm^3^. Although the data obtained at 152 °C are of limited reference value because of severe foam deformation at the excessively high foaming temperature, the SEM micrographs still show a consistent trend of increasing cell size with increasing EPR content.

The increase in cell size and decrease in cell density are mainly attributed to the regulation of CO_2_ dissolution and diffusion behavior by EPR. As an amorphous rubber phase, EPR increases the CO_2_ solubility and diffusion rate of the blends, acting as both a CO_2_ reservoir and a preferential pathway for CO_2_ diffusion [28]. Upon rapid depressurization, the system becomes highly supersaturated. In the presence of EPR, dissolved CO_2_ can be rapidly transported through the EPR-rich phase toward the initially formed cell nuclei, promoting their growth into larger cells. Meanwhile, rapid long-range CO_2_ migration reduces the local supersaturation in the surrounding matrix, thereby suppressing the formation of new nuclei and decreasing the overall cell density. In addition, the cellular structure of neat PP foamed at 148 °C was too poor and nonuniform for reliable statistical analysis.

The decrease in cell density with increasing EPR content should therefore be interpreted by considering the combined effects of cell nucleation, CO_2_ transport, and melt viscoelasticity. Although dispersed EPR domains may provide additional interfaces for heterogeneous cell nucleation, the CO_2_ desorption results indicate that EPR incorporation also increases CO_2_ solubility and accelerates gas diffusion. Similar phenomena have been reported for PP-based multiphase foams, where a dispersed CO_2_-affinitive phase can promote CO_2_ accumulation and enhance the diffusion rate of CO_2_ in the PP matrix, thereby strongly affecting the balance between cell nucleation and cell growth [29]. Therefore, the reduced cell density observed in the PP/EPR bead foams is more likely associated with the enhanced gas transport and preferential growth of existing cells, rather than simply with a decrease in the number of potential nucleation sites.

In addition, the rheological results show that EPR incorporation increases the low-frequency viscoelastic response of the blends. Previous studies on PP foaming have shown that insufficient melt strength and melt elasticity can lead to cell wall rupture, cell coalescence, and foam collapse, whereas enhanced melt viscoelasticity helps stabilize growing cells [4,12]. In the present system, the increased melt viscoelasticity may help resist cell wall thinning and suppress cell collapse during bead expansion. Thus, the enlarged but relatively stable closed-cell structure of PP/EPR bead foams can be attributed to the coupled effects of enhanced CO_2_ transport and improved melt viscoelasticity. Nevertheless, when the EPR content is excessive, as in the 30 wt% blend, the co-continuous EPR-rich phase may cause rapid gas loss and unstable cell growth, which is unfavorable for obtaining a uniform cellular structure [4].

### 3.7. Melting Behavior of PP/EPR Bead Foams

**Figure 10:**
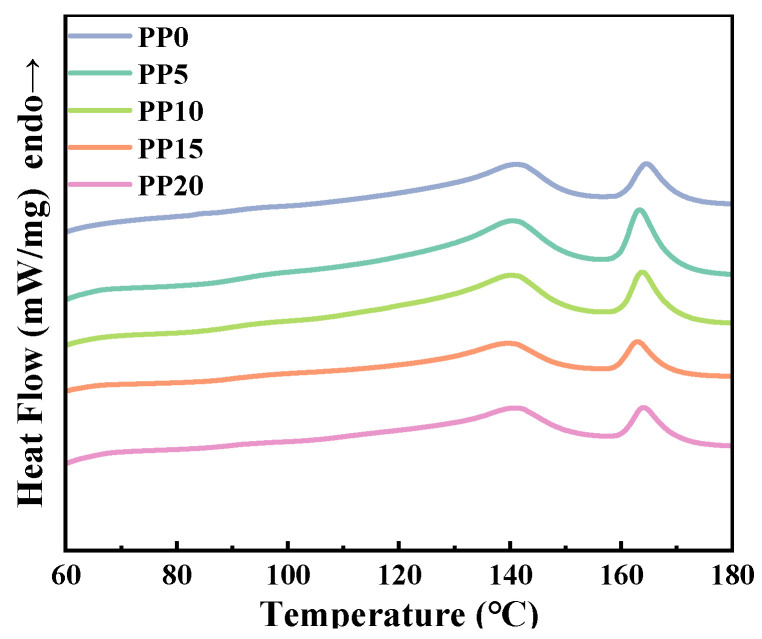
DSC curves of different PP/EPR bead foams.

The DSC melting curves and the corresponding double-melting peak parameters of PP/EPR bead foams are shown in Figure 10 and Table 6, respectively. Because inter-bead adhesion and shape retention during steam-chest molding largely depend on the unique double-melting behavior of foam beads, it is necessary to investigate the effect of EPR content on their melting behavior using DSC [30,31,32]. The DSC results show that both the total melting enthalpy and the low-temperature melting enthalpy of the bead foams increase significantly with the incorporation of 5 wt% and 10 wt% EPR. This can be attributed to the relatively long ethylene segments in the EPR molecular chains, which may act as heterogeneous nucleation sites in the PP matrix and induce the formation of imperfect crystals with thinner lamellae and lower thermal stability. The increased amount of these imperfect crystals directly raises the proportion of the low-temperature melting fraction.

However, as the EPR content further increases, the dilution effect of the amorphous rubber phase and the steric hindrance arising from intensified chain entanglement become dominant. This restricts the ordered packing of PP segments, weakens the overall crystallization ability of the blends, and consequently reduces the melting enthalpy.

This variation in thermal behavior has a dual positive effect on the molding performance of bead foams. On the one hand, the high-temperature melting peak remains stable at around 164 °C, corresponding to thermodynamically stable and more perfect crystals. During steam heating, these crystals serve as a rigid framework within the beads, preventing cell collapse and maintaining the macroscopic shape stability of the molded products. On the other hand, the enhanced low-temperature melting fraction indicates that more imperfect crystals, particularly those near the bead surface, can melt under the same steam pressure. During steam-chest molding, the crystals corresponding to the low-temperature melting peak of EPP beads can melt selectively, providing mobile PP chains at the bead surface and promoting chain interdiffusion across adjacent bead interfaces [33]. Since the strength of a thermally welded polymer interface depends strongly on interfacial chain diffusion and the formation of entanglements across the interface [34], the increased low-temperature melting enthalpy in the PP/EPR bead foams is beneficial for inter-bead welding. Together with the viscoelastic EPR phase near the bead interface, the molten PP chains can enhance interfacial diffusion and chain entanglement, thereby improving the welding strength between beads.

### 3.8. Mechanical Properties of PP/EPR Bead Foam Products

**Figure 11:**
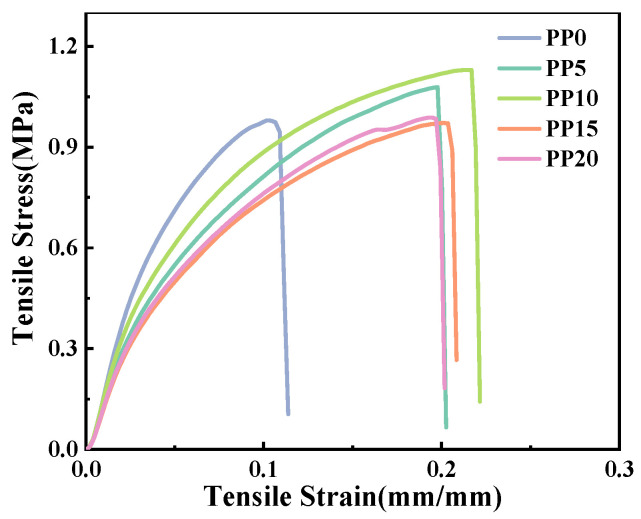
Tensile stress–strain curves of PP/EPR bead foam samples.

As shown in Figure 11 and Table 7, PP/EPR bead foam samples with similar densities were tested to evaluate the effect of EPR content on the tensile performance of molded products prepared under identical conditions. Owing to the toughening effect of the rubber phase, the elongation at break of all EPR-modified bead foams is significantly improved, increasing from 11.4% for neat PP to more than 20%. The tensile strength first increases and then decreases with increasing EPR content, reaching a maximum value of 1.13 MPa at 10 wt% EPR, compared with 0.98 MPa for neat PP.

In addition to the intrinsic strength of the matrix, the tensile properties of foam materials are strongly governed by their cellular structure, and foams with finer and more uniform cells generally exhibit better tensile performance. However, unlike conventional one-step foaming processes, bead foam molding involves pre-pressurization followed by steam heating to produce the final molded products. Therefore, for bead foam products, inter-bead welding quality plays a crucial role in determining the final tensile properties, in addition to the contribution of the cellular structure.

EPR incorporation increases the cell size of the pre-foamed beads. Larger cells may facilitate air penetration and secondary expansion during the pre-pressurization stage, thereby promoting more effective secondary expansion in the steam chest. This increases the compressive contact force between adjacent beads and consequently improves inter-bead adhesion. In addition, because EPR is an amorphous elastomer without a distinct melting point, the dispersed EPR phase can act as a binder at the bead interfaces. As discussed in Section 3.2 and Section 3.7, EPR incorporation restricts ordered chain packing, refines the crystallite domains, and promotes the formation of imperfect PP crystals. This structural modification reduces the thermal stability of the low-temperature melting fraction and increases the low-temperature melting enthalpy of the bead foams. Under the same molding steam pressure, more imperfect crystals can melt and participate in interfacial diffusion and chain entanglement, thereby enhancing inter-bead welding and improving the tensile properties of the final molded products [35].

Although EPR improves the tensile properties of the molded products by enhancing inter-bead adhesion, its weakening effect on the PP matrix and the influence of cell size variation on mechanical performance cannot be ignored [36]. When the EPR content exceeds 10 wt%, the positive contribution of improved inter-bead adhesion is gradually outweighed by the reduced load-bearing capacity of the PP matrix. Therefore, the tensile strength is effectively improved at moderate EPR contents, especially at 10 wt%. However, with further increasing EPR content, the matrix-weakening effect becomes dominant, leading to a gradual decline in tensile strength.

As shown in Table 8, the enhanced inter-bead adhesion induced by EPR enables the modified bead foams to achieve tensile properties comparable to those of neat PP even at lower molding steam pressures. This indicates that EPR incorporation can reduce the steam pressure required for steam-chest molding, thereby decreasing energy consumption and overall production costs.

**Figure 12:**
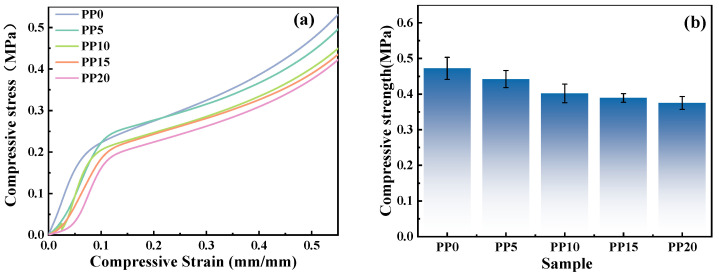
Compressive properties of PP/EPR bead foam blocks: (**a**) compressive stress–strain curves and (**b**) compressive strength at 50% deformation.

Unlike the tensile properties, the compressive properties are less affected by the improvement in inter-bead adhesion. As shown in Figure 12, the cell-enlarging effect induced by EPR leads to a certain deterioration in compressive performance [36,37]. However, unlike conventional foams that rely primarily on cell walls for load bearing, bead foams also contain outer bead shells that can support part of the compressive load. As a result, the negative effect of cell enlargement on compressive performance is partially mitigated.

## 4. Conclusions

This study demonstrates that EPR incorporation provides an effective route for regulating the foaming behavior and steam-chest molding performance of PP bead foams. The dispersed EPR phase modifies the crystalline morphology, CO_2_ transport behavior, and melt viscoelasticity of the PP matrix, thereby broadening the processing window and improving the stability of cell growth during bead foaming. The role of EPR is not limited to conventional rubber toughening. In the PP/EPR blends, EPR acts simultaneously as a CO_2_-rich phase and a preferential gas-transport pathway, which promotes the growth of existing cells while reducing excessive cell nucleation. Meanwhile, the enhanced melt elasticity helps stabilize the enlarged cellular structure. These coupled effects provide a mechanistic basis for controlling the cell structure of PP-based bead foams. EPR also contributes to the steam-chest molding behavior of the bead foams by regulating the double-melting characteristics and promoting interfacial chain diffusion between adjacent beads. At an appropriate EPR content, the improved inter-bead welding compensates for the reduced intrinsic strength of the PP matrix and leads to enhanced tensile performance of the molded foam products. However, excessive EPR weakens the load-bearing PP phase and may deteriorate the balance between cell structure and mechanical properties.

Overall, PP/EPR bead foams combine improved foamability, enhanced inter-bead adhesion, and reduced molding steam-pressure requirements, indicating their potential for recyclable lightweight molded products such as protective packaging, automotive energy-absorbing components, and reusable logistics applications.

## Figures and Tables

**Table 1:** Formulations of PP/EPR blends.

Sample	PP (wt%)	EPR (wt%)	Antioxidant (phr)
PP0	100	0	0.1
PP5	95	5	0.1
PP10	90	10	0.1
PP15	85	15	0.1
PP20	80	20	0.1
PP30	70	30	0.1

**Table 2:** Thermal parameters of PP/EPR blends.

Sample	*T_m_*/°C	*T_c_*/°C	*X_c,app_*/%	*X_c,PP_*/%
PP0	148	108	24.5	24.5
PP5	148	109	24.4	25.7
PP10	148	108	24.1	26.8
PP15	147	109	23.4	27.5
PP20	147	109	22.1	27.6
PP30	147	108	21.0	30.0

**Table 3:** Crystallite sizes of different crystal planes.

Crystal Planes	(110)	(040)	(130)	(111)/(131)
PP0	121 Å	223 Å	208 Å	155 Å
PP5	120 Å	222 Å	205 Å	155 Å
PP10	105 Å	223 Å	180 Å	143 Å
PP15	106 Å	218 Å	183 Å	144 Å
PP20	104 Å	214 Å	194 Å	141 Å
PP30	102 Å	222 Å	210 Å	150 Å

**Table 4:** Desorption diffusion coefficients of CO_2_ in PP and PP/EPR blends saturated at 25 °C and 5 MPa.

Sample	M_0_ (g CO_2_/100 g Polymer)	*D_d_* (×10^−10^ m^2^/s)
PP0	3.55	5.39
PP5	4.00	5.40
PP10	4.44	5.53
PP15	4.90	5.57
PP20	5.35	5.75
PP30	5.77	8.06

**Table 5:** Expansion ratios of bead foams at different temperatures.

Sample	PP0	PP5	PP10	PP15	PP20
148 °C	7.9	8.9	9.1	15.3	10.2
150 °C	18.7	20.9	21.1	19.7	20.3
152 °C	32.1	27.3	27.5	24.8	26.2

**Table 6:** Double-melting peak parameters of PP/EPR bead foams.

Sample	Low-Temperature Melting Enthalpy (J/g)	Low-Temperature Peak (°C)	High-Temperature Melting Enthalpy (J/g)	High-Temperature Peak (°C)
PP0	28.5	141	11.1	165
PP5	36.8	140	15.2	163
PP10	37.8	140	12.7	164
PP15	25.3	140	8.3	163
PP20	28.6	141	9.4	164

**Table 7:** Tensile properties of PP/EPR bead foams.

Sample	Elongation at Break/%	Tensile Strength/MPa	Tensile Modulus/MPa	Steam Pressure/Bar
PP0	11.37 ± 0.91	0.98 ± 0.08	18.77 ± 1.50	2.80
PP5	20.25 ± 1.62	1.08 ± 0.06	12.89 ± 1.03	2.80
PP10	22.17 ± 1.77	1.13 ± 0.04	14.61 ± 1.17	2.80
PP15	20.81 ± 1.66	0.97 ± 0.07	11.86 ± 0.95	2.80
PP20	20.19 ± 0.53	0.99 ± 0.06	12.01 ± 0.96	2.80

**Table 8:** Tensile properties of PP/EPR bead foams molded under different steam pressures.

Sample	Elongation at Break/%	Tensile Strength/MPa	Tensile Modulus/MPa	Steam Pressure/Bar
PP0	11.37 ± 0.91	0.98 ± 0.08	18.77 ± 1.50	2.80
PP5	15.86 ± 0.74	1.02 ± 0.05	15.79 ± 1.26	2.60
PP10	12.22 ± 1.32	0.95 ± 0.07	17.24 ± 1.38	2.40
PP15	19.93 ± 1.27	1.06 ± 0.04	15.64 ± 1.25	2.60
PP20	18.39 ± 1.59	1.00 ± 0.03	14.19 ± 1.14	2.40

## Data Availability

The original contributions presented in this study are included in the article/Supplementary Material. Further inquiries can be directed to the corresponding author.

## Supplementary Materials

The following supporting information can be downloaded at: https://www.mdpi.com/article/10.3390/polym18121540/s1, Table S1: Formulations of PP/EPR blends for preliminary batch foaming experiments; Figure S1: SEM images of PP/EPR foams prepared by preliminary batch foaming of compression-molded sheets.; Figure S2: Expansion ratios of compression-molded PP/EPR sheets prepared by preliminary batch foaming at different temperatures; Table S2: Cellular parameters of compression-molded PP/EPR sheets after preliminary batch foaming at 135 °C.
