# Supplementary material for: Cell Structure Regulation of Polypropylene/Ethylene-Propylene Rubber Bead Foams and Enhanced Mechanical Properties of Their Molded Products
Source: Polymers (Basel). 2026 Jun 21;18(12):1540. doi: 10.3390/polym18121540 (PMC13306762; doi:10.3390/polym18121540)
Supplement: Supplementary file 1 [file polymers-18-01540-s001.zip › polymers-4382276-supplementary.pdf]

Supplementary material

Cell Structure Regulation of Polypropylene/Ethylene-Propylene Rubber Bead Foams and Enhanced Mechanical Properties of Their Molded Products

2. Experimental section

2.1 Sample preparation for preliminary batch foaming experiments

PP/EPR blends were processed using a HAKKE torque rheometer (HAKKE Polylab 557–1306) at a processing temperature of 180 °C and a rotor speed of 50 rpm for 5 min. Detailed blending information and naming can be found in Table S1.

Table S1. Formulations of PP/EPR blends for preliminary batch foaming experiments.

Sample	PP（phr）	EPR（phr）	Antioxidant（phr）
PP0	100	0	0.1
PP10	90	10	0.1
PP20	80	20	0.1
PP30	70	30	0.1

The dried samples were hot-pressed into circular sheets with dimensions of 35 mm × 2 mm for intermittent foaming experiments. The modified PP discs were placed in the mold cavity, passed through a low-pressure CO_2 purge 3 times, pressurized to the desired CO_2 pressure (15MPa), and saturated at a set foaming temperature (130 °C, 133 °C, 135 °C). After 45 min, the intermittent foaming samples were obtained with a pressure reduction rate of about 80MPa/s.

3. Results and discussion

3.1 Preliminary batch foaming behavior of compression-molded PP/EPR sheets

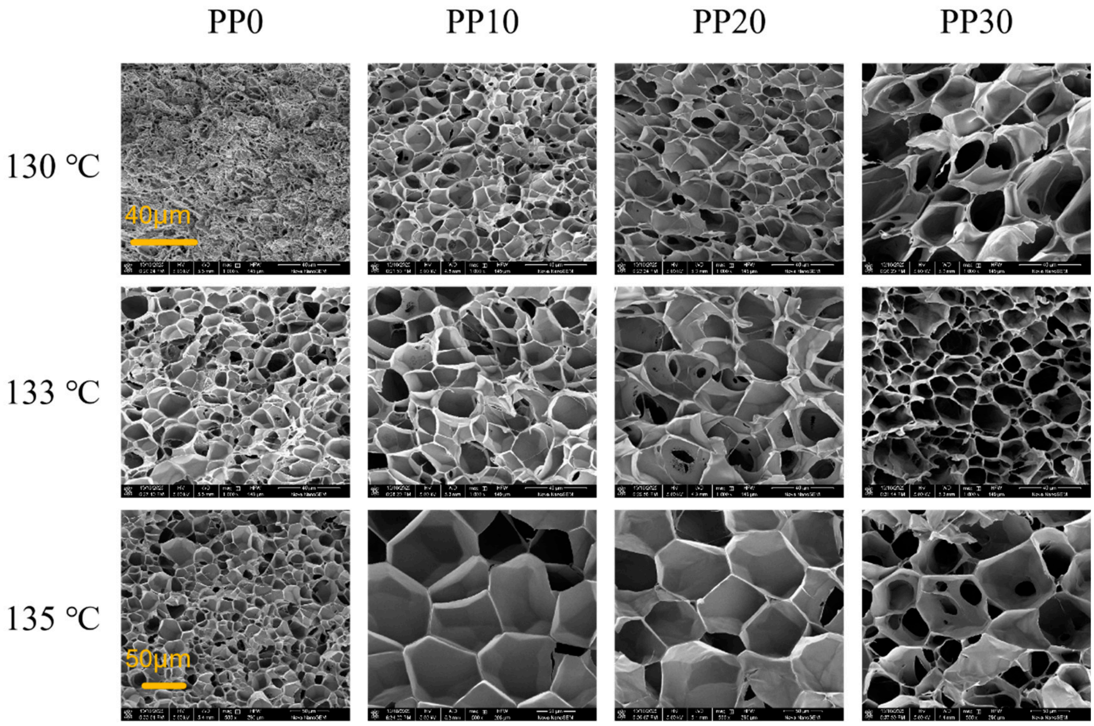

Figure S1. SEM images of PP/EPR foams prepared by preliminary batch foaming of compression-molded sheets.

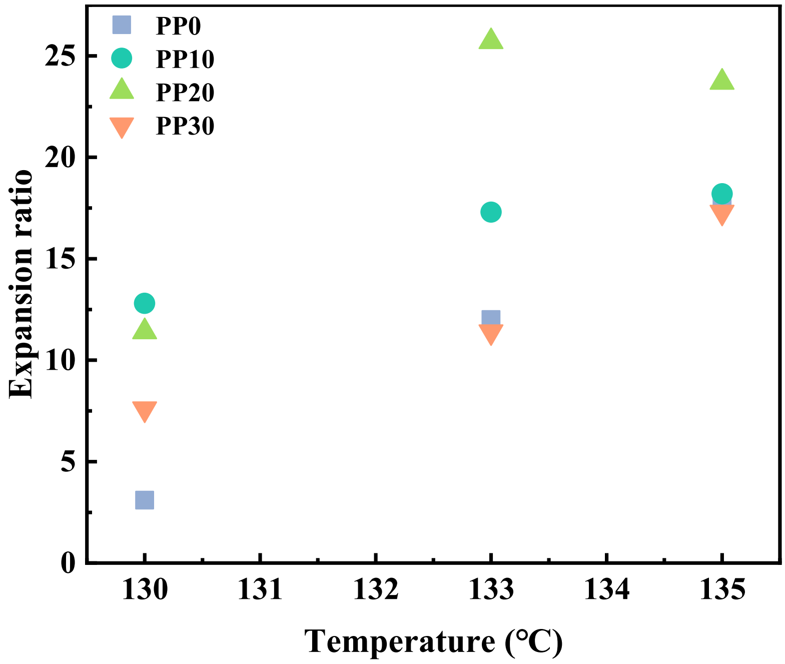

Figure S2. Expansion ratios of compression-molded PP/EPR sheets prepared by preliminary batch foaming at different temperatures.

Table S2. Cellular parameters of compression-molded PP/EPR sheets after preliminary batch foaming at 135 °C.

Sample	cell diameter(μm)	cell density（cells/cm^3）
PP0	10.7	4.00×10^{11}
PP10	62.3	8.90×10^{10}
PP20	62.5	8.50×10^{10}
PP30	49.9	1.24×10^{11}
